# The Adsorption of Arsenate and Arsenite Ions on Oxidic Substrates Prepared with a Variable-Charge Lithological Material

**DOI:** 10.3390/ma17225544

**Published:** 2024-11-13

**Authors:** Xinyao Ren, Enju Wang, Fernando Millán, José G. Prato, Marin Senilă, Andrés Eduardo Márquez Chacón, Luisa Carolina González, Guido P. Santillán Lima, Carla Silva Padilla

**Affiliations:** 1 Department of Chemistry, Saint John’s University, Jamaica, NY 11439, USA; xyyren307@gmail.com (X.R.); wange@stjohns.edu (E.W.); 2 Ingeniería Química, Instituto Universitario Politécnico “Santiago Mariño”, Mérida 5101, Venezuela; 3 Grupo de Investigación Estudios Interdisciplinarios, Facultad de Ingeniería, Universidad Nacional de Chimborazo, Av. Antonio José de Sucre km 1½ vía Guano, Riobamba 060103, Ecuador; psantillan@unach.edu.ec (G.P.S.L.); carla.silva@unach.edu.ec (C.S.P.); 4 Ingeniería Química, Facultad de Ingeniería, Universidad de Los Andes, Mérida 5101, Venezuela; 5 Research Institute for Analytical Instrumentation, INCDO INOE 2000, Donath 67, RO-400293 Cluj-Napoca, Romania; marin.senila@icia.ro; 6 Instituto de Investigación, Facultad de Farmacia y Bioanálisis, Universidad de Los Andes, Mérida 5101, Venezuela; andresmqch@gmail.com; 7 Grupo de Investigación “Análisis de Muestras Biológicas y Forenses”, Laboratorio Clínico, Facultad de Ciencias de la Salud, Universidad Nacional de Chimborazo, Av. Antonio José de Sucre km 1½ vía Guano, Riobamba 060103, Ecuador; luisacarolinagonzalez@gmail.com; 8 Laboratorio de Investigaciones Parasitológicas “Jesús Moreno Rangel”, Cátedra de Parasitología, Departamento de Microbiología y Parasitología, Facultad de Farmacia y Bioanálisis, Universidad de Los Andes, Mérida 5101, Venezuela

**Keywords:** adsorption, arsenic, arsenate ions, isotherm, lithological materials, water treatment

## Abstract

The adsorption of As(V) and As(III) (0.01–1 mM) on a calcined oxidic lithologic material substrate with pH-dependent surface variable charges, chemically modifiable, was investigated. The substrate was prepared via thermal treatment using a natural lithologic material rich in amphoteric oxides of Fe, Al, Mn and Ti. The calcined substrate was treated with acid media (HCl 0.1) to homogenize the positive charge density on the oxide surface via oxide protonation so that anion adsorption would be favored. A batch experiment was performed on the acid-treated substrate (activated) and non-activated substrate. L-type isotherms were obtained, which fit the Freundlich model. Isotherm constants showed that there was a greater affinity between the activated substrate and As(V) (*K* = 10.58) compared to As(III) (*K* = 5.45). The adsorption capacity of the activated substrate was two times greater than that of the non-activated substrate, As(V) (*K_act_* = 10.58 and *K_noact_* = 5.45) vs. As(III) (*K_act_* = 5.45 y *K_noact_* = 2.44), which was due to the greater positive charge density on the activated surface, created by the protonation of the surface oxides. Protons were liberated during the adsorption reaction (As(V): 2.17 × 10^−3^ and As(III): 0.96 × 10^−3^ mmol/mL). The forms H_2_AsO_4_^−^ and H_3_AsO_3_ deprotonated when adsorbed by the surface groups $M-OH_{2}^{+}$ (M: Fe, Al). Kinetic data showed a second-order process for As(V) adsorption and a first-order process for As(III) adsorption. The adsorption rate on the activated substrate was two times greater compared with the non-activated substrate: As(V) (*k_act_* = 3.78 × 10^−5^ L/mg·min and *k_noact_* = 2.16 × 10^−5^ L/mg·min) vs. As(III) (*k_act_* = 0.055 h^−1^ and *k_noact_* = 0.027 h^−1^). The tested substrate is potentially useful as a low-cost natural material for arsenic removal from contaminated water.

## 1. Introduction

Water filtration and purification are critical topics of discovery and exploration. Intensive agricultural production and many industrial and mining activities, as well as natural processes such as meteorization and volcanism, generate diverse toxic pollutants responsible for water contamination. One of these pollutants is arsenic, the 20th most abundant element in nature [1,2,3]. The Earth’s crust contains 1.5–2 mg/kg As in the inorganic forms, arsenate As(V) and arsenite As(III), both being very toxic and having enough solubility and mobility to be transported to water systems [3,4,5]. Arsenic can be mobilized into the natural environment through different mechanisms such as weathering, biological activity, volcanism, and leaching, among others. Arsenic can be present in coal as arsenopyrite in various quantities; American coal may contain up to 71 mg/kg As [6]. There are many geographic zones in the world that are seriously affected by the presence of native arsenic in natural water [3,4]. According to the literature, 350,000 km^2^ in the United States are affected by this problem, particularly in the states of Nevada, California, and Arizona, where natural water could contain up to 2.6 mg/L As [7,8]. However, according to the USEPA and the WHO recommendations, the concentration of arsenic in drinking water is set by law to be below 10 μg/L. Thus, extensive arsenic removal is critically needed to achieve this standard in many places [2,3,4,5,6].

In Latin America, almost all countries are affected by the presence of natural arsenic in drinkable water above the permissible level. An estimation of 14 million people are at risk of poisoning. Many of these areas are rural communities where traditional technologies are not available or they are not justified economically. This fact forces the application of technologies with great artisanal components, using available natural materials useful for water treatment through adsorption processes [9].

The symptoms of arsenic poisoning might appear after a long time, when it is often too late for a cure. Arseniosis or Regional Chronic Endemic Hidroarsenisism might look like bruises or excoriations on the skin; it might also manifest as neuronal, gastric, or hematologic effects, including a whole series of cancerous pathologies [10].

Many physical, chemical, and biological processes have been applied for arsenic (and other pollutants) removal from water, such as chemical precipitation, flocculation, reverse osmosis, and ion exchange. However, most of these technologies have serious disadvantages such as high costs, the production of dangerous waste products, and low efficiency. Because of these drawbacks, adsorption processes have been adopted for water treatment because they offer certain advantages. They are more efficient, environmentally friendly, adaptable, and versatile due to the great variety of adsorbent materials that can be used [11,12,13,14,15]. Synthetic activated carbon is a good As adsorber. Its holding capacity will depend on the source and method of production [11,14]. Natural zeolites, thermally modified, have been tested for heavy metal adsorption from drinkable water with good results [16,17]. However, their effectiveness may vary based on specific surface modifications [16,17]. Red mud has been described in the literature for As adsorption [18]. Despite its good potential as an adsorbent for inorganic forms of arsenic removal, red mud is classified as a hazardous material because of its caustic and saline nature.

In search of new natural adsorbent materials for heavy metal adsorption that are low in cost, non-toxic, environmentally friendly, and readily available, researchers have tested a new kind of adsorbent substrate based on a natural oxidic and refractory lithologic material for heavy metal adsorption [19,20,21]. “Lithologic materials” are all those materials belonging to the Earth’s crust that cannot be classified as soil. Oxidic lithologic materials are mainly composed of refractory amorphous iron, aluminum, manganese, and titanium oxides, as well as amorphous aluminosilicates [21,22,23,24]. Their chemical and physical characteristics have been reported in the literature [19,24,25,26]. The main characteristics of these oxides are their high thermal resistance and their large oxidic surface with variable electrical charges that are pH-dependent [25,26,27,28,29]. According to Equation (1), in an alkaline medium, oxides deprotonate, which increases the negative charge density on the substrate surface, allowing for cationic adsorption. On the contrary, in an acid medium, oxides protonate and create a greater positive charge density on the substrate surface, promoting anionic adsorption [19,22].

(1) $$M-O^{-}\overset{+OH^{-}}{\leftarrow }M-{OH}^{0}\overset{+H^{+}}{\to }M-{OH}_{2}^{+}$$

X-ray absorption analysis [30,31,32] showed the formation of both inner-sphere monodentate and bidentate binuclear of As(V) complexes on ferrihydrite and goethite. John et al. [13] reported that on iron oxide adsorbents, the adsorption of As(V) at neutral pH occurs through the formation of monodentate at low surface coverage. At high surface coverage, As(V) is adsorbed through the formation of bidentate complexes because it can occupy two adsorption sites at the same time. Therefore, the coverage of the positive charge density on the substrate surface is important for the adsorption mode. In addition, the computational simulation performed by Dzade and Leeuw [31] suggests that arsenic anionic forms, i.e., arsenate and arsenite, bond to an Fe-OH surface in the ferrihydrite mineral structure via binuclear–bidentate complexation or inner-sphere complexes. These structures were shown to be more thermodynamically stable than monodentate–mononuclear or outer-sphere complexes. The reported adsorption energy for the binuclear–bidentate complex (−3.39 eV) is 35% lower than that of the mononuclear–monodentate complex (−2.23 eV).

In Latin America, over half of the population resides in small, rural communities that often lack sufficient economic and material resources. These areas face ongoing challenges related to heavy metal contamination in water, which is an increasingly pressing issue given the limitations in access to advanced water treatment systems. The high costs and infrastructure requirements of these conventional systems render them economically unfeasible for many rural communities, where official support may be limited or sporadic.

Oxidic lithologic materials (OLMs), commonly available across the region, offer a promising and underutilized resource for addressing water contamination in these vulnerable settings. These materials have been used for preparing calcined substrates with surfaces chemically modified to achieve anion adsorption [24,26] and cation adsorption [22,23,24,25,29]. Leveraging such locally sourced materials to develop low-cost, accessible, and sustainable solutions for water treatment holds the potential to enhance water quality significantly and reduce the health risks associated with arsenic exposure. This project emphasizes sustainability and equity, seeking to promote simple, effective, and economically viable technologies that improve the quality of life in rural communities and foster greater local autonomy.

The present study, therefore, focuses on understanding the adsorption efficiency of chemically treated oxidic lithologic surfaces in removing anionic forms of As(III) and As(V) from water, aiming to provide a practical, sustainable approach for resource-limited regions.

## 2. Materials and Methods

### 2.1. Chemicals and Standard Solutions

Potassium arsenate, monobasic (KH_2_ArO_4_, 99%), and sodium meta arsenite (NaAsO_2_, 90%) for (As(III)) were obtained from Sigma-Aldrich, St. Louis, MI, USA. A 10 mM (749 ppm As) solution of KH_2_AsO_4_ was prepared with distilled–deionized water, while lower concentrations of 1 to 0.01 mM (74.9 to 0.75 ppm As) were prepared with serial dilutions and these solutions were used for the As(V) adsorption experiments. Similarly, solutions of NaAsO_2_ prepared at the same concentrations were used for the As(III) adsorption experiments.

### 2.2. Preparation of the Adsorbent Substrate

**Figure 1:**
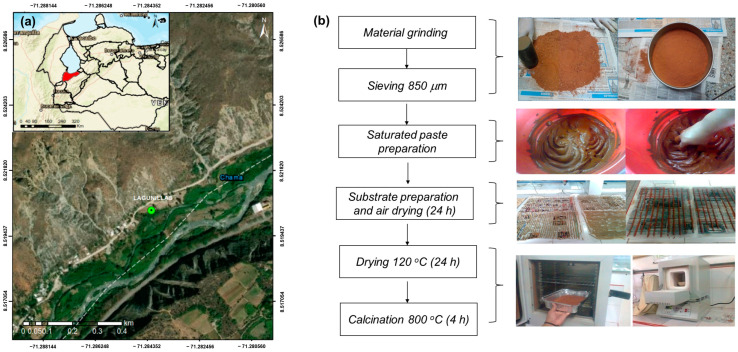
(**a**) Geographic localization. (**b**) Schematic representation of experimental procedure.

A 5 kg sample of the 8°28′47″ N and 71°23′47″ W, located north west of Mérida city, Mérida State, Venezuela (Figure 1a). It is an arid zone, where the temperature ranges between 17 and 30 °C over the course of year, with a maximum rainfall of 200 mm per year. The place is a mine used by potters to prepare bricks via thermal treatment. The lithologic material has been previously characterized and the results have been reported in the literature [19,25,26]: a brick-red sandy loam material with a relatively low exchange capacity (13.4 cmol/kg) and very low organic material content (0.40%). Al (11.75%), Fe (7.24%), Ti (0.37%), and Mn (0.03%) are the major metals found in the oxidic lithological material and they are present as refractory amphoteric oxides [25]. The alkali and alkaline earth contents are Na (1%) and K (1.62%), and Ca (0.032%), Mg (0.31%), Ba (0.04%), and Sr (0.005%) [19]. Several traces of transition metals are also present.

The granulometric fraction smaller than 800 μm was separated using an 800 μm laboratory sieve (ASTME II Endecotts Ltd., London, UK) coupled to an automatic vibrator (Octagon Digital CE) for 15 min. Above this particle size, the sand percentage increases and deteriorates the mechanical properties of the pellet. This procedure has been described in the literature [25]. After grinding and sieving, a hand-made soft saturated paste was prepared with distilled water. Cylindrical strips of 3–4 mm in diameter were extruded through a 60 mL syringe, cut into 5–6 mm long pellets, and air-dried for 24 h, followed by drying in a furnace at 120 °C for 12 h and finally calcination in a muffle at 800 °C for 5 h (Figure 1b). The hard red cylinders were cooled in the muffle to room temperature and then stored in a sealed container before use. The calcination process completely burns out the organic matter; therefore, only the mineral phase is involved in the adsorption reaction. Calcination also favors oxide formation and the cementation of the pellets, avoiding its dispersion in the solution.

The specific surface area and average pore volume of the calcined materials were determined through N_2_ adsorption–desorption measurements conducted at −196 °C. Prior to these measurements, the materials were subjected to heat treatment at 400 °C under vacuum for 12 h. The analysis was performed using a Micromeritics ASAP 2420 Surface Area and Porosity Analyzer (Micromeritics Instrument Corporation, Norcross, GA, USA). Macroporosity was determined by water saturation, according to the method described by Foth [33].

### 2.3. Activation of the Substrate

The purpose of the activation procedure was to increase the positive charge density on the oxidic surface so that it favors anion adsorption. The calcined substrate was chemically treated in acid media with 0.1 M HCl solution for 12 h at room temperature; during that time, a protonation reaction of the amphoteric oxides took place according to Equation (1). After this time, the substrate was washed with distilled water until neutral pH was achieved and then dried in the furnace at 120 °C for 12 h. The product was then called the activated substrate.

### 2.4. Adsorption Studies

Adsorption experiments were performed on both the activated and non-activated substrate in triplicate under isothermal conditions at 20 ± 0.1 °C for 24 h using a batch equilibration procedure. We treated seven samples of 2 g of the activated and non-activated substrate with 20 mL solution of increasing As(V) and As(III) concentration (0.01 to 1.0 mM) in closed containers. The solutions were initially mixed for 10 min at 50 rpm and placed in a thermostatic oven at 20 ± 0.1 °C for 24 h, with periodical manual agitation. Arsenic equilibrium concentration was measured with an optical ICP emission spectrometer, Thermo Scientific ICP 7000 series (Thermo Fisher Scientific Inc., Waltham, MA, USA), at 189.42 nm and 193.759 nm. Table 1 shows the linear calibration functions in the range from 1 to 1000 ppm As and the correlation coefficients with the standard As(V) solutions.

pH and electrical conductivity (EC) were measured during the adsorption reaction. pH was measured with a Thermo Scientific Orion STAR A111 pH-meter (Waltham, MA, USA) previously calibrated with buffer solutions of pH 4 and 7. EC was measured with a Thermo Scientific Orion STARTM A112 conductivity meter (Waltham, MA, USA) previously calibrated with a saline solution of concentration 692 mg/L (1413 mS/cm). Typically, 2 g of the substrate and 20 mL of As(V) solution were stirred together; then, the pH electrode or the conductometer was placed in the mixture and the pH or the EC values were continuously monitored for 60 min.

Kinetic studies were performed using the batch procedure with six sets of 2 g of the substrate and 20 mL of 45 ppm As(V) or As(III) solutions under stirring. The adsorption was stopped at different times (1–60 min) for each set. The As concentration in the contact solution was filtered and prepared for the ICP-OES measurement.

The adsorption isotherms were obtained by plotting the amount of As adsorbed against the equilibrium concentration in the solution and fitted with the Freundlich model, shown in Equation (2), and the logarithmic form of the Freundlich equation, shown in Equation (3) [12,29]:

(2) $$\left(\frac{x}{m}\right)=K\;{\left(C_{\mathrm{eq}}\right)}^{1/n}$$

(3) $$L\mathrm{og}\left(\frac{x}{m}\right)=\mathrm{Log}\left(K\right)+\frac{1}{n}\mathrm{Log}\left(C_{\mathrm{eq}}\right)$$

where $\frac{x}{m}$ is the ratio between the amount of adsorbed species and the amount of the adsorbent, and *K* and 1/n are constants which represent the adsorption capacity and the adsorption intensity, respectively. By plotting Log(x/m) vs. Log C_eq_, a straight line should be obtained, whose linear correlation coefficient *r* can be quantified. Significant linearity was tested according to the significance statistical test for the *r* coefficient and the coefficient of determination *R*^2^ [34]. The *K* constant (adsorption capacity) was given by the intercept, and the n value was calculated from the slope of the straight line.

## 3. Results and Discussion

### 3.1. Specific Surfaces and Macroporosity

The substrate pellet had an average volume of 26.25 ± 5.18 mm^3^ with an RSD of 19.75% and a density of 1.70 g/mL. Such high variability in the volume was due to the hand-made process of preparing the substrate. However, this fact did not affect the adsorption process. Table 2 shows the values of the specific surface area and macroporosity of the calcined substrate.

The specific surface has a relatively low value compared to Kaolinite of low crystallinity (50 m^2^/g) [35,36]. However, the pore volume and pore width were large, and more solution can be stored in them.

According to Lombardi et al. [37], the specific surface area could be underestimated because the N_2_ adsorption method presents accuracy limitations in charged surfaces with a specific surface area of around 10 m^2^/g. The N_2_ molecule is a non-polar molecule and does not have total access to the adsorption active sites in the charged surface like a polar molecule like water would.

Macro and mesoporosity refer to the empty space that can be filled by water displacing air, corresponding to the pores with a size greater than 2 nm. Because of the high surface tension of water, it is difficult to access microporosity, i.e., those pores with a size smaller than 2 nm. Thus, the trapped air there cannot be evacuated. The macro and mesoporosity of the substrate represent nearly one-third of the solid volume, and the average pore size (74.60 Å) of the substrate is greater than 2 nm. This fact favors the penetration of the aqueous solution.

### 3.2. As(V) Adsorption

#### 3.2.1. Adsorption Isotherms

**Figure 2:**
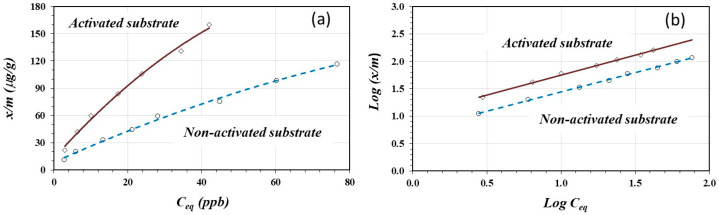
Adsorption isotherms (**a**) and Freundlich model linear fitting (**b**) for As(V) on activated and non-activated sorbents.

Figure 2a shows the adsorption isotherms for As(V) on the acid-activated and non-activated substrates. Both isotherms can be classified as *L*-type isotherms which could be indicative of chemisorption. The graphs suggest that the adsorbate has greater affinity on the activated substrate. According to Adamson [38], in the Freundlich equation, at low concentrations, the x/m vs. the C_eq_ does not become linear, but remains convex to the concentration axis, without showing a saturation zone. However, chemisorption must occur in the first monolayer so that physical adsorption may occur above the chemisorbed layer.

Figure 2b shows the linear fittings according to the Freundlich model expressed by Equation (3). Both linear fittings show good linearity with a statistically significant correlation for α = 0.01 [34]. Table 3 shows the linear-form equations, the coefficient of determination “*R*^2^”, the constant *K*, and the *n* values for the activated and non-activated substrates.

The As(V) adsorption capacity, given by the constant *K*, was two times greater on the acid-activated substrate compared to the adsorption on the non-activated substrate, as shown in Table 3. This can be interpreted as a more favorable condition for As(V) adsorption on the acid-activated surface due to the protonation reaction of the oxidic groups, according to Equation (1). The protonation reaction during acid treatment creates a greater positive charge density on the oxidic surface. A higher positive charge surface coverage provides more available sites for the adsorption reaction to occur. On the contrary, the adsorption reaction on the non-activated substrate is less favorable due to the limited availability of adsorption sites.

Joshi et al. [15] reported a similar *K* value (9.18) for As(V) adsorption on an iron oxide/activated carbon substrate to that of As(V) adsorption on the activated oxidic substrate (10.58). However, their n value is greater on pure iron oxide/activated carbon substances.

Similar adsorption capacities were obtained when using protonated adsorbents such as nano-sized cerium oxide, iron oxide nanoparticles, and oxide-modified activated carbon composites, with the yield varying between 6 and 13 depending on the conditions of activation, the metal content, and the surface characteristics [14,39,40,41].

**Figure 3:**
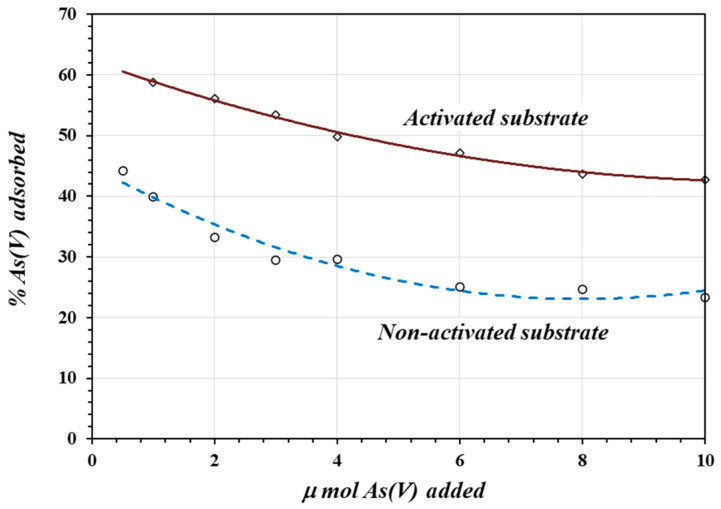
The percentage of As(V) adsorbed as a function of *μ* mole of As(V) added to the solution.

Figure 3 shows the percentage of As(V) adsorbed as a function of the amount of As(V) added to two grams of acid-activated and non-activated substrates. On average, the adsorption of As(V) on the acid-activated substrate is about two times greater than on the non-activated substrate. In both cases, at a lower number of moles of As(V) (lower concentration in the contact solution), there is a higher percentage of adsorption on 2 g of the substrate. At higher concentrations, more ions compete for the limited active adsorption sites and saturate the adsorption surface, resulting in a lower percentage of adsorption.

The variable-charge property of this material has been proved by Marquez et al. [23], who determined the zero-charge point (ZCP) in the raw material as well as on calcined activated and non-activated surfaces. Marquez’s experiment showed that the calcination process favors oxide formation, and the surface charge is pH-dependent. The greater adsorption of As(V) on the activated surface shown in Figure 3 could be due to the increased positive charge on the oxidic surface through protonation during the acid activation process according to the reaction in (1), which favors anion adsorption.

Similar results have been reported for the adsorption of As(V) on zeolites, iron oxide-coated sand, and alumina [42,43,44], where the adsorption efficiency on acid-activated materials was significantly higher compared to on non-activated adsorbents, attributing the increased performance to the higher positive charge density created during the acid activation process.

#### 3.2.2. pH and Electrical Conductivity Variations

**Figure 4:**
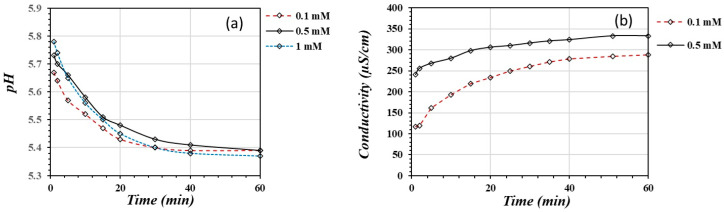
The variation in pH (**a**) and EC (**b**) throughout the adsorption reaction of As(V) on the activated substrate.

Figure 4 shows the variation in pH (a) and EC (b) throughout the adsorption reaction of As(V) on the activated substrate. The adsorption reaction takes place with the production of protons within the first 20 min; after this time, the system tends to reach equilibrium. Acidification is equivalent to 2.17 × 10^−3^ μmoles/mL H^+^ produced during the adsorption reaction.

The pH varies within a range from 5.8 to 5.4. This pH range lies within the pH (3.0–8.0) range for maximum As(V) adsorption on the iron oxide/activated carbon substrate [15]. Within this pH range, anionic forms of As(V) are more stable and more susceptible to adsorption onto the oxidic substrates. The As forms which are thermodynamically stable under oxidizing conditions at this pH value are H_2_AsO_4_^−^ (*p*K_a1_ = 2.19) between pH 2 and 6, and HAsO_4_^−2^ (*p*K_a2_ = 6.94) at pH greater than 6 [14,45]. Therefore, the main species of As(V) present in the solutions is H_2_AsO_4_^−^. This form may release one proton when chemically adsorbed by the positively activated sites on the oxidic surface according to Equation (4). Therefore, the dissociated protons cause solution acidification.

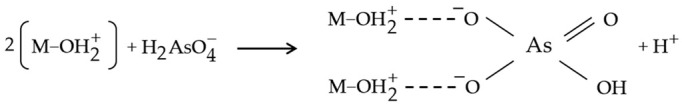
(4)

Equation (4) suggests that As(V) binds to protonated hydroxyl groups in a bidentate complex after dissociating a proton from the orthoarsenate form. Some authors agree with the notion that arsenic adsorption on iron-based materials takes place through the formation of mono- or bidentate inner-sphere complexes [5,14,15]. Hao et al. [45] suggest the formation of bidentate inner-sphere complexes for As(V) adsorption on iron-based adsorbents. Also, Mojiri et al. [14] reported the formation of the stable bidentate binuclear surface complex on metal oxide nano-adsorbents, such as titanium oxide. Therefore, Equation (4) is a possible reaction between solid iron oxy-hydroxides and the orthoarsenate ion through the formation of a binuclear bidentate surface complex. The complexation reaction liberates protons, so the pH decreases and the EC increases.

Figure 4b shows the variation in electrical conductivity (EC) during the adsorption reaction. The conductivity meter detected an increase in the ionic activity in the solution in the first 20 min. After this time, the system tended to reach equilibrium. These results suggest that the protons produced in the adsorption reaction are responsible for the EC increase.

#### 3.2.3. Kinetic Measurements

**Figure 5:**
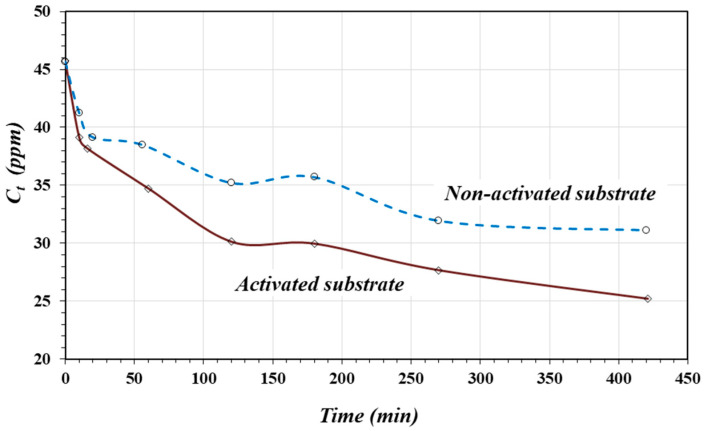
As(V) adsorption kinetics on activated and non-activated substrate.

Figure 5 shows the As(V) adsorption kinetics on the activated and non-activated substrates. The adsorption reaction was a fast process for the first 20 min, especially on the activated substrate. After this time, the adsorption rate decreased, and equilibrium was reached after two hours of the reaction.

However, Figure 5 suggests that the adsorption reaction takes place in two steps with different speeds. During the first 20 min, the surface was almost saturated because of the formation of the first monolayer, which was chemically adsorbed. The formation of the second layer, which was physically adsorbed, was much slower because of the different kinds of interactions between the adsorbed molecules and the first monolayer. Joshi et al. [15] applied a second-order kinetic to describe the adsorption of As(V) onto iron oxide/activated carbon.

Considering the adsorption process as second order, the rate equation is defined by the expression in (5):

(5) $$-\frac{\mathrm{dC}}{\mathrm{dt}}=k\times C^{2}$$

The variables must be separated and integrated on both sides of the equation:

(6) $$-\int_{C_{0}}^{C}\frac{dC}{C^{2}}=k\times \int_{0}^{t}dt$$

Finally, this results in a linear function expressed by Equation (7):

(7) $$\frac{1}{\;\;C}=\frac{1}{C_{0}}+k\times t\;$$

**Figure 6:**
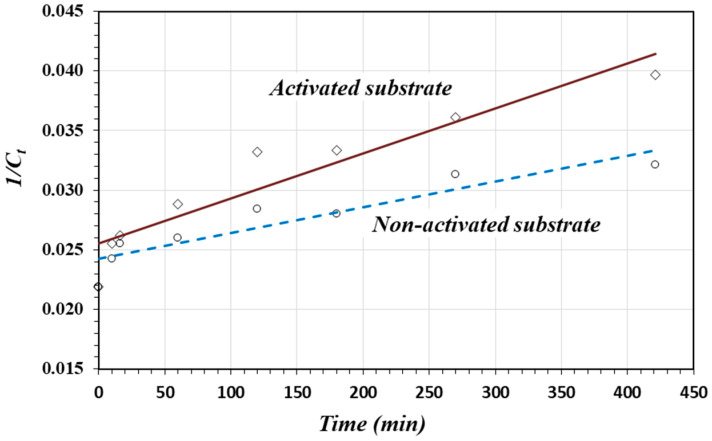
Linear plots for the second-order model for As(V) adsorption on the activated and non-activated substrates.

By plotting $\frac{1}{C}\;vs.\;t$, the rate constant *k* can be calculated from the slope of the straight line. Figure 6 shows the linear curves for the adsorption of As(V) on the activated and non-activated substrates, and Table 4 presents the respective equations, as well as the *k* and *R*^2^ values. The adsorption rate (*k* value) on the activated substrate was two times greater than the adsorption rate on the non-activated substrate.

This experimental fact can be easily explained based on Equation (1). The oxide protonation reaction creates a greater positive charge density on the surface of the activated substrate. Consequently, the adsorption rate increases. On the non-activated substrate, the smaller positive charge density makes the adsorbate molecules interact and compete for the very limited adsorption sites; thus, the adsorption rate is smaller and the surface saturates faster.

### 3.3. Adsorption of As(III)

#### 3.3.1. Adsorption Isotherms

**Figure 7:**
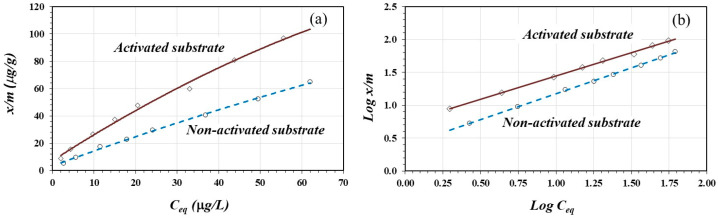
Isotherms (**a**) and Freundlich fitting equation (**b**) for As(III) adsorption on activated and non-activated substrates.

Figure 7a shows the adsorption isotherms for As(III) on the activated and non-activated substrates. Similar to the case of As(V) adsorption, both isotherms might be classified as *L*-type isotherms and could indicate chemisorption. In the same way, the activated substrate had a greater affinity for As(III) compared to the non-activated substrate. Figure 7b shows the linear fittings according to the Freundlich model expressed by Equation (3). Both linear fittings show good linearity with a statistically significant correlation for α = 0.01. Table 5 shows the linear-form equations, the coefficient of determination “*R*^2^”, the constant *K,* and the *n* values for the activated and non-activated substrates.

As shown in Table 5, the constant *K* (adsorption capacity) for As(III) adsorption on the acid activated substrate was two times greater than that on the non-activated substrate. The *n* values (adsorption intensity) suggest more favorable adsorption of As(III) on the activated substrate. This is a consequence of the protonation reaction in the surface oxides. As in the case of As(V) adsorption, a greater positive charge density represents a greater probability for the adsorption reaction to occur. However, the *K* values are larger for As(III) adsorption compared to using Fe_2_O_4_/nano-porous carbon (*K* = 2.29) [15].

The adsorption capacities of As(V) and As(III) depend on the preparation conditions, activation, metal content, and surface characteristics of the adsorbents. When using activated carbons, the yields can vary between 0.03 and 51.3 mg/g [14,41,46], while when using modified zeolites, the adsorption capacity varies between 0.05 and 2.8 mg/g [42,47].

**Figure 8:**
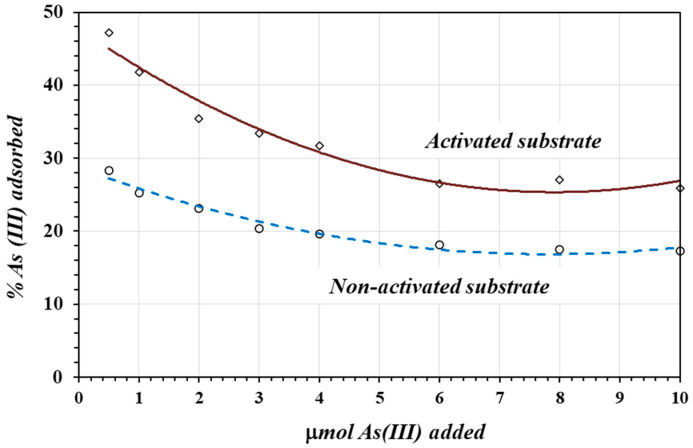
The percentage of As(III) adsorbed as a function of *μ* mole of As(III) added to the solution.

Figure 8 shows the percentage of As(III) adsorbed as a function of the amount of As(III) added to samples of activated and non-activated substrates. Here, again in both cases, at a lower number of moles of As(III), there was a higher percentage of adsorption. At higher concentrations, the adsorbing molecules saturate the surface. The adsorption of As(III) on the activated substrate was about 1.5 to 2 times greater than on the non-activated substrate.

#### 3.3.2. pH and Electrical Conductivity Variations

**Figure 9:**
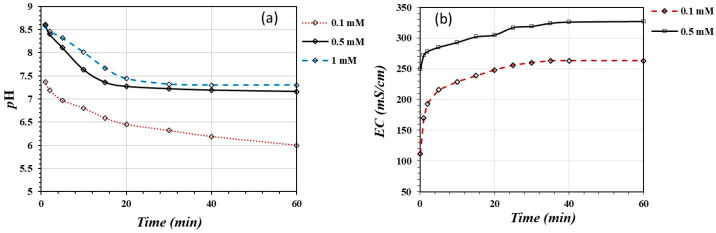
Changes in pH (**a**) and electrical conductivity (EC) (**b**) during adsorption reaction of As(III) on activated and non-activated substrates.

Figure 9a shows the variation in pH throughout the reaction for three different concentrations of As(III). The adsorption reaction takes place relatively fast during the first 20 min with the production of protons, especially at lower concentrations of As(III). In this case, 9.60 × 10^−4^ mmol H^+^/mL was produced. This amount of protons was more than two times less than in the case of the As(V) adsorption reaction due to a lower affinity of the oxidic substrate for As(III) anionic species.

The pH varied within a range from 8.5 to 6. The thermodynamically stable form of As(III), under moderate oxidizing conditions at these pH values, is mainly H_3_AsO_3_ (*p*K_a1_ = 9.2) and a small amount of H_2_AsO_3_^−^ (*p*K_a2_ = 12.7). The deprotonation of H_3_AsO_3_ to orthoarsenite, the H_2_AsO_3_^−^ form, may occur during absorption onto the oxidic surface containing $M-OH_{2}^{+}$ positively charged sites. The dissociated protons cause solution acidification according to Equation (8):

(8) $$M-OH_{2}^{+}+H_{3}AsO_{3}\to M-OH_{2}^{+}-O-As{\left(OH\right)}_{2}+H^{+}$$

Equation (8) suggests that As(III) might bind to protonated hydroxyl groups through a monodentate complex after dissociating a proton from the orthoarsenite form. Joshi et al. [15] suggested a similar mechanism for As(III) adsorption on iron oxide nano-porous carbon composites, and Hao et al. [45] also suggested the formation of monodentate inner-sphere complexes for As(V) adsorption on iron-based adsorbents.

Figure 9b shows the variation in electrical conductivity throughout the adsorption reaction. The Conductimeter detected an increase in the ionic activity in the solution during the first 20 min of the reaction. After this time, EC measurements became relatively constant. Similar to the As(V) adsorption reaction, most probably the EC increased due to proton generation during the adsorption reaction.

#### 3.3.3. Kinetic Studies

**Figure 10:**
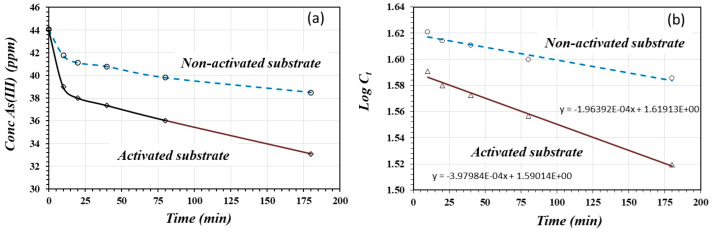
As(III) adsorption kinetics on activated and non-activated substrates (**a**), and linear functions for first-order model for activated and non-activated substrate (**b**).

Figure 10 shows the As(III) adsorption kinetics on the activated and non-activated substrates (a), as well as the fitting equations for the first-order kinetic model (b). As in the case of the As(V) adsorption reaction, As(III) adsorbed quickly in the first 20 min, especially on the activated substrate. After this time, the adsorption rate decreased on both the non-activated and activated substrates.

Figure 10a suggests that the adsorption reaction of As(III) takes place in one single step, since the first-order kinetic model fits the data better for the adsorption of As(III) on the adsorbent substrate. In this case, the rate equation is defined by Equation (9):

(9) $$-\frac{dC}{dt}=k\times C$$

The variables can be separated and integrated on both sides of the equation:

(10) $$-\int_{C_{0}}^{C}\frac{dC}{C}=k\times \int_{0}^{t}dt$$

This results in a linear function expressed by Equation (11):

(11) $$logC=logC_{0}-\frac{k}{2.303}\times t$$

By plotting log⁡C vs. t, the rate constant *k* can be calculated from the slope of the straight line (Figure 10b). Table 6 presents the respective equations and the *k* and *R*^2^ values for the activated and non-activated substrates. In both cases, the coefficient *R*^2^ is favorable for linear fitting, especially for the adsorption of As(III) on the activated substrate where the adsorption rate was two times greater in relation to adsorption on the non-activated substrate. The greater positive charge density created more available sites for anion adsorption.

## 4. Conclusions

The main objective of the present paper was to investigate the relative affinity between the oxidic calcined substrate with variable charges and the As(V) and As(III) ionic species in aqueous media. The results show the great affinity between adsorbates and the substrates. The activated substrate particularly favored the adsorption of the H_2_ASO_4_^−^ and H_2_AsO_3_^−^ ionic species due to the protonation reaction of the amphoteric oxides in acid media, which enlarges the positively charged density on the oxidic surface. The adsorption isotherms fit the *L*-type Freundlich model, which is associated with specific adsorption or chemisorption. The adsorption reaction of As(V) and As(III) produced solution acidification, especially on the activated substrate. Protons were produced when H_2_ASO_4_^−^ and H_3_AsO_3_ deprotonated due to being adsorbed by the surface groups M-OH_2_^+^. The adsorption reaction probably took place through chemisorption with the formation of bidentate or monodentate complexes with the terminal groups –OH_2_^+^, depending on the surface coverage of the positively charged density. The greater adsorption capacity on the activated surface is in agreement with the theory stated in Equation (1). This makes the substrate potentially applicable for arsenic retention from polluted waters. Oxidic lithological materials are widely available and inexpensive, and the substrates are easily made. These characteristics make them suitable to be used in rural environments where traditional procedures of water treatment cannot be implemented or are not economically justifiable. Such substrates could also be useful for the removal of other dangerous metallic species to produce safe water.

Future studies are essential to further enhance the adsorption capacity of these oxidic lithologic materials, optimizing their application for the treatment of natural waters in rural communities. Additional research could focus on the modification of surface properties to improve selectivity and efficiency not only for arsenic but also for other hazardous contaminants commonly present in these environments. Such investigations would support the development of practical, low-cost water treatment solutions tailored to rural settings, providing a sustainable alternative for safe drinking water and expanding the applicability of these substrates to a broader range of pollutants.

## Figures and Tables

**Table 1:** Trend lines for calibration graph for As at 189.042 and 193.754 nm.

α (nm)	Equation	*R* ^2^
189.042	EI = −0.2555 + 0.1295 × C_AS_	0.9998 *
193.754	EI = −0.3425 + 0.1250 × C_AS_	0.9998 *

* Significant linearity for α = 0.01. EI is emission intensity.

**Table 2:** Specific surface area and macroporosity of calcined substrate.

Specific Surface (m^2^/g)	33.82
Pore volume (μL/g)	140.06
Pore width (Å)	165.65
Pore size (Å)	74.60
Macroporosity (%)	29.39

**Table 3:** Freundlich fitted equations for As(V) adsorption on activated and non-activated substrates.

Substrate	Fitted Equation	*R* ^2^	*K*	*n*
Activated	Log(x/m) = 1.0246 + 0.7253 × Log(C_eq_)	0.9962 *	10.58	1.38
Non-activated	Log(x/m) = 0.7364 + 0.7019 × Log(C_eq_)	0.9982 *	5.45	1.42

* Significant linearity for α = 0.05 (activated) and α = 0.01 (non-activated) substrates.

**Table 4:** Second-order equations for As(V) adsorption on activated and non-activated substrates.

Substrate	Fitted Equation	*R* ^2^	*k* (L/mg · min)
Activated	1/C = 0.0255 + 3.78 × 10^−5^ × t	0.8860 *	3.78 × 10^−5^
Non-activated	1/C = 0.0243 + 2.16 × 10^−5^ × t	0.8565 *	2.16 × 10^−5^

* Significant linearity for α = 0.05 (activated) and α = 0.01 (non-activated) substrates.

**Table 5:** Freundlich fitted equations for As(III) adsorption on activated and non-activated substrates.

Substrate	Fitted Equation	*R* ^2^	*K*	*n*
Activated	Log(x/m) = 0.7362 + 0.7085 × Log(C_eq_)	0.9978 *	5.45	1.41
Non-activated	Log(x/m) = 0.3875 + 0.7865 × Log(C_eq_)	0.9992 *	2.44	1.27

* Significant linearity for α = 0.05 (activated) and α = 0.01 (non-activated) substrates.

**Table 6:** First-order kinetic model and *k* values.

Substrate	Fitted Equation	*R* ^2^	*k* (min^−1^)
Activated	log*C_eq_* = 1.5961 − 4.002 × 10^−4^ × *t*	0.9882 *	9.22 × 10^−4^
Non-activated	log*C_eq_* = 1.6191 − 1.997 × 10^−4^ × *t*	0.9602 *	4.60 × 10^−4^

* Significant linearity for α = 0.05 (activated) and α = 0.01 (non-activated) substrates.

## Data Availability

The original contributions presented in the study are included in the article, further inquiries can be directed to the corresponding authors.
